# New and Paradoxical Roles of Matrix Metalloproteinases in the Tumor Microenvironment

**DOI:** 10.3389/fphar.2012.00140

**Published:** 2012-07-17

**Authors:** Agnès Noël, Ana Gutiérrez-Fernández, Nor Eddine Sounni, Niels Behrendt, Erik Maquoi, Ida K. Lund, Santiago Cal, Gunilla Hoyer-Hansen, Carlos López-Otín

**Affiliations:** ^1^Laboratory of Tumor and Development Biology, GIGA-Cancer, University of LiègeLiège, Belgium; ^2^Departamento de Bioquímica y Biología Molecular, Instituto Universitario de Oncología, Universidad de OviedoOviedo, Asturias, Spain; ^3^The Finsen Laboratory, Copenhagen University Hospital, Copenhagen BiocenterCopenhagen N, Denmark; ^4^Biotech Research & Innovation Centre, University of Copenhagen, Copenhagen BiocenterCopenhagen N, Denmark

**Keywords:** matrix metalloproteinases, ADAM, cancer, tumor, microenvironment

## Abstract

Processes such as cell proliferation, angiogenesis, apoptosis, or invasion are strongly influenced by the surrounding microenvironment of the tumor. Therefore, the ability to change these surroundings represents an important property through which tumor cells are able to acquire specific functions necessary for tumor growth and dissemination. Matrix metalloproteinases (MMPs) constitute key players in this process, allowing tumor cells to modify the extracellular matrix (ECM) and release cytokines, growth factors, and other cell-surface molecules, ultimately facilitating protease-dependent tumor progression. Remodeling of the ECM by collagenolytic enzymes such as MMP1, MMP8, MMP13, or the membrane-bound MT1-MMP as well as by other membrane-anchored proteases is required for invasion and recruitment of novel blood vessels. However, the multiple roles of the MMPs do not all fit into a simple pattern. Despite the pro-tumorigenic function of certain metalloproteinases, recent studies have shown that other members of these families, such as MMP8 or MMP11, have a protective role against tumor growth and metastasis in animal models. These studies have been further expanded by large-scale genomic analysis, revealing that the genes encoding metalloproteinases, such as MMP8, MMP27, ADAM7, and ADAM29, are recurrently mutated in specific tumors, while several ADAMTSs are epigenetically silenced in different cancers. The importance of these proteases in modifying the tumor microenvironment highlights the need for a deeper understanding of how stroma cells and the ECM can modulate tumor progression.

## Introduction

Genetic alterations in tumor cells are essential for tumor development but not sufficient to generate malignant tumors. The tumor stroma resulting from an evolving crosstalk between tumor cells and different host cell types is required to create a permissive environment for the invasion of genetically altered tumor cells (Hanahan and Weinberg, 2011; Lu et al., 2012). Key modifications of the stromal environment include enhanced vascularization following an “angiogenic switch” (Bergers et al., 2000), quantitative and qualitative changes in the extracellular matrix (ECM), and the recruitment of resident fibroblastic cells (Kalluri and Zeisberg, 2006), bone marrow-derived mesenchymal stem cells (Spaeth et al., 2009) and inflammatory cells (Coussens and Werb, 2001). The importance of the tumor microenvironment is now recognized as fundamental for cancer progression (Joyce and Pollard, 2009), but the critical molecular changes occurring in the tumor stroma accompanying and affecting cancer evolution remain largely unknown. Desmoplasia, the fibrotic stromal reaction associated with most carcinomas, is characterized by the local deposition of fibrillar collagen types I, III, and V. This host reaction correlating with adverse prognosis in mammary carcinomas (Hasebe et al., 2002) is also seen in metastatic sites (Erler and Weaver, 2009). Remarkably, increased expression of interstitial collagen and many of its remodeling enzymes is frequently detected in gene signatures associated with poor prognosis in cancer patients (Ramaswamy et al., 2003; Finak et al., 2008; Tavazoie et al., 2008). In addition to quantitative changes in collagen deposition, the architecture of the collagen scaffold is also drastically affected during cancer evolution. In this context, collagen crosslinking by lysyl oxidase (LOX) whose expression is increased upon hypoxic conditions has emerged as a key determinant of late stage tumors (Erler et al., 2009).

It is now recognized that proteinases contribute actively to the elaboration of the stromal microenvironment during early and late stages of primary and secondary tumor development (Holmbeck et al., 2003; Noel et al., 2008). The degradation of collagen by cathepsins and matrix metalloproteinases (MMPs), and the receptor-mediated endocytosis of degraded collagen are important events that regulate cancer cell survival, growth, migration, and invasion. Proteinases act not only by disrupting physiological barriers to ease cell migration, but importantly by releasing growth and chemotactic factors from the ECM and unmasking cryptic domains of matrix components (Lopez-Otin and Overall, 2002; Kalluri, 2003). In addition, these enzymes are key regulators of shedding, activation, and/or degradation of cell-surface molecules including adhesion molecules, mediators of apoptosis, receptors of chemokines/cytokines, and intercellular junction proteins (Overall and Kleifeld, 2006; Cauwe et al., 2007; Lopez-Otin and Hunter, 2010).

In this review, we focus on secreted metalloproteinases (MMPs and disintegrin-metalloproteinases with thrombospondin domains, referred to as ADAMTSs) and the associated cell-surface receptor (uPARAP/endo180) specifically involved in interstitial collagen remodeling. We also describe novel findings generated by the collaborative EU-FP7 funded network, MicroEnviMet (No. HEALTH-F2-2008-201279). This project has shed light on novel functions of membrane-associated MMPs in the control of cell apoptosis and angiogenesis, as well as on the complex tumor-host interplay in which proteinases can either boost cancer progression or protect the host against malignancy.

## MMPs and Related Enzymes

**Figure 1:**
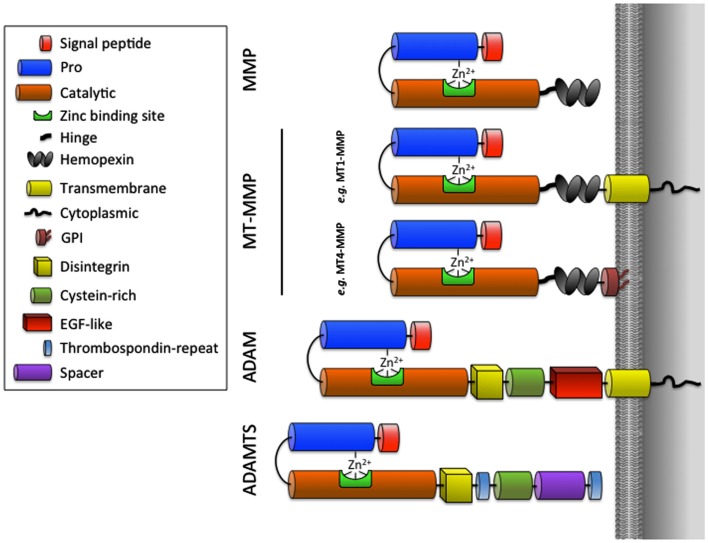
**Schematic representation of MMPs, MT-MMPs, ADAMs, and ADAMTSs**.

It is now recognized that proteinases contribute to all stages of tumor progression (growth, angiogenesis, invasion, and evasion to immune system) and are produced not only by the tumor cells themselves, but mainly by the different non-malignant host cells composing the tumor. Among the different classes of proteinases implicated during different stages of cancer progression, the MMPs constitute a family of 24 human zinc-binding endopeptidases that can degrade virtually all ECM components and have a growing number of substrates belonging to all important families of cell regulators: integrins, cell-surface receptors, kinases, chemokines, and cytokines (Egeblad and Werb, 2002; Lopez-Otin and Overall, 2002; Folgueras et al., 2004; Overall and Kleifeld, 2006; Cauwe et al., 2007; Lopez-Otin and Hunter, 2010). Most MMPs are secreted as soluble enzymes but six of them are membrane-type MMPs (MT-MMPs) that are associated with the cell membrane by either a COOH-terminal transmembrane domain (MT1-, MT2-, MT3-, MT5-MMP) or a glycosylphosphatidyl-inositol (GPI) anchor (MT4- and MT6-MMP). For a description of the structure, function, and regulation of MMPs and MT-MMPs, the reader is referred to previous reviews (Zucker et al., 2003; Sounni and Noel, 2005; Page-McCaw et al., 2007; Sohail et al., 2008; Fanjul-Fernandez et al., 2010; Kessenbrock et al., 2010; Strongin, 2010). The ADAMs are membrane-anchored proteinases that share the catalytic domain with the MMPs but which include two main differences: (1) the absence of a hemopexin-like domain and (2) the insertion of three additional domains [cysteine-rich domain, epidermal growth factor (EGF)-like domain and the disintegrin domain; Figure 1; Klein and Bischoff, 2011]. The related ADAMTS family contains 19 human metalloproteinases with a variable number of type-1 thrombospondin (TSP-1) domains in their C-terminal region. ADAMTSs are now viewed as key regulators of collagen maturation (ADAMTS-2, -3, and -14; Colige et al., 2005; Dubail et al., 2010), cartilage degradation (ADAMTS-1, -4, -5, -8, and -9), microfibril biogenesis (Hubmacher and Apte, 2011), von Willebrand factor maturation (ADAMTS-13), reproduction (ADAMTS-9, -20; Llamazares et al., 2007), and cancer progression (Handsley and Edwards, 2005; Rocks et al., 2008).

## Collagen Remodeling

The fibrillar collagens (e.g., types I, II, III) are composed of three polypeptides α-chains (homotrimers or heterotrimers) assembled into a triple-helical structure forming the collagenous domain. The N-terminal non-collagenous domain of these fibrillar collagens is proteolytically removed by ADAMTS-2 (Dubail et al., 2010). Interstitial collagenases are the only known mammalian enzymes able to degrade triple-helical fibrillar collagens through specific cleavage of all three α-chains at a single locus three-quarters from the N-terminus. Collagenolytic MMPs include soluble MMPs (MMP1, MMP8, MMP13) and the membrane-associated MMP14/MT1-MMP, MMP15, and MMP16. More recently, MMP2 has been identified as an interstitial collagenase that can cleave native type I collagen in a distinctive way from other collagenases without generating the classical 3/4 and 1/4 fragments (Egeblad et al., 2007). The very similar gelatinase, MMP9, does not cleave collagen but shares gelatinolytic activity with MMP2 (Vihinen et al., 2005).

Interestingly, microarray analyses have identified collagenolytic MMP1 in a gene expression signature able to predict distant metastasis in breast cancer patients (van’t Veer et al., 2002; Gupta et al., 2007). Moreover, MMP1 appears to be a key determinant that selectively mediates lung metastasis in a murine breast cancer model (Minn et al., 2005, 2007; Nguyen and Massague, 2007). MMP13 (collagenase-3) originally identified in human breast cancer tissue (Freije et al., 1994) is viewed as a potential tumor marker for breast cancer diagnosis (Chang et al., 2009) and its expression is correlated with metastasis formation (Ellsworth et al., 2009; Lee et al., 2009). In experimental models, MMP13 appears as a key stromal mediator of cancer progression that regulates the release of angiogenic factors (Lederle et al., 2009) and metastatic dissemination (Zigrino et al., 2009). Interestingly, the presence of microinvasion in ductal carcinoma *in situ* (DCIS) is associated with focal expression of *MMP13* mRNA in stromal fibroblasts (Nielsen et al., 2001, 2007). However, in the aggressive mouse mammary tumor virus-polyoma middle T-antigen (MMTV-PyMT) model of breast cancer, the absence of MMP13 did not influence tumor growth, vascularization, or metastasis to the lungs, suggesting that the role of MMP13 in breast cancer may depend on the nature of the genetic lesions driving malignancy (Nielsen et al., 2008).

MT1-MMP (MMP14) has emerged as an important collagenase that cancer cells use to degrade and invade in a collagen-rich environment (Poincloux et al., 2009; Sabeh et al., 2009). *Mmp14^−/−^* mice exhibit skeletal defects with craniofacial abnormalities, osteopenia, and impaired angiogenesis (Holmbeck et al., 1999; Zhou et al., 2000). These mutant mice are the unique *Mmp*-deficient mice generated up to now that are associated with a severe phenotype leading to death after birth. Type I collagen cleavage by MT1-MMP at the endothelial cell-surface stimulates migration, guidance, and organization of endothelial cells into tubular structures (Collen et al., 2003). In the tumor microenvironment, type I collagen remodeling by MT1-MMP enables cancer cells to escape the mechanical barriers confined by the collagen matrix, and stimulates tumor growth *in vivo* (Hotary et al., 2003). We have recently demonstrated that while poorly invasive breast adenocarcinoma cells undergo apoptosis when confronted with a collagen-rich environment, the production of MT1-MMP endows these cells with the capacity to escape from collagen-induced apoptosis (Maquoi et al., 2012). Beyond its well known gelatinolytic functions, MMP2 also displays interstitial collagenolytic activity (Egeblad et al., 2007) that unexpectedly contributes to lymphangiogenesis, the formation of new lymphatic vessels (Detry et al., 2011). The other gelatinase, MMP9, plays a critical role in tumor-induced angiogenesis through release of vascular endothelial growth factor (VEGF) sequestered from the ECM (Bergers et al., 2000).

In addition to this MMP-driven collagen degradation process, separate pathways, mediated by cysteine protease cathepsins, are operative in acidic extracellular or intracellular microenvironments. The intracellular pathway involves the binding of collagen fibrils to specific cell-surface receptors followed by the cellular uptake and proteolytic degradation of internalized collagen in the lysosomal compartment. One such receptor is uPARAP/Endo180, a member of the macrophage mannose receptor family of endocytic transmembrane glycoproteins. This receptor plays a key role in the cellular uptake and lysosomal degradation of collagen fragments generated through the initial MMP-mediated collagen cleavage (Kjoller et al., 2004; Curino et al., 2005; Engelholm et al., 2009). In cell lines, the amount of internalized collagen correlates with the levels of uPARAP expression (Madsen et al., 2007, 2011). The genetic ablation of uPARAP/Endo180 in mice demonstrated that the uPARAP-driven endocytic route of collagen breakdown is a rate-limiting factor in collagenolysis by fibroblastic cells, chondrocytes, and osteoclasts (Engelholm et al., 2003; Kjoller et al., 2004; Sulek et al., 2007), as well as in collagen turnover in fibrosis (Bundesmann et al., 2012; Lopez-Guisa et al., 2012; Madsen et al., 2012) and in the invasive growth of breast tumors in mice (Curino et al., 2005). Notably, uPARAP regulates the autolysis and cell-surface level of MT1-MMP reinforcing the functional interplay between two collagen degradation pathways (Kogianni et al., 2009; Messaritou et al., 2009).

## Pro-Tumorigenic Functions of MT-MMPs

Beside its role in tumor cells, MT1-MMP is recognized as a crucial regulator of angiogenesis in collagen- or fibrin-rich environments (Chun et al., 2004; Stratman et al., 2009). MT1-MMP’s pro-angiogenic capacities in both physiological and pathological conditions are related to several mechanisms including: (1) ECM remodeling (Hotary et al., 2003), (2) interaction with cell-surface molecules, such as CD44 (Kajita et al., 2001) and sphingosine 1-phosphate (S1P; Langlois et al., 2004), (3) degradation of anti-angiogenic factors such as decorin in cornea (Mimura et al., 2009), or (4) interaction with TIMP-2 and signaling through ERK1/2 during cell migration (Sounni et al., 2010b). In addition, MT1-MMP plays a role in transcriptional and posttranslational control of VEGF expression and bio-availability (Deryugina et al., 2002; Sounni et al., 2002, 2004; Eisenach et al., 2010), as well as in hematopoietic progenitor cell mobilization (Vagima et al., 2009), due to so far unknown molecular mechanisms. Furthermore, a number of recent reports have shed light on an important interplay between MT1-MMP and TGFβ during angiogenesis and vessel maturation (Tatti et al., 2008; Hawinkels et al., 2010; Sounni et al., 2010a, 2011).

In contrast to MT1-MMP, MT4-MMP is unable to activate proMMP2. Furthermore, MT4-MMP is rather inefficient in hydrolyzing most ECM components compared to the other MT-MMPs (Zucker et al., 2003). Its catalytic domain is able to cleave very few substrates *in vitro*, including gelatin, fibrin(ogen), lipoprotein receptor-related protein, proTNF-alpha, and the aggrecanase ADAMTS-4 (Sohail et al., 2008). The largely overlooked functions of the GPI-anchored MT4-MMP have been explored by the MicroEnviMet partners. In human breast cancer samples, a higher intensity of MT4-MMP immunostaining is observed in cancer cells compared to normal breast epithelial cells (Chabottaux et al., 2006). The overexpression of MT4-MMP in the breast cancer cell line MDA-MB-231 enhances subcutaneous tumor growth and most importantly leads to lung metastasis when cells are inoculated in RAG-1 immunodeficient mice (Chabottaux et al., 2006, 2009). The pro-metastatic effect of MT4-MMP is dependent on its proteolytic activity (Chabottaux et al., 2006) and relies on the induction of an early angiogenic switch (Host et al., 2012) and the perturbation of blood vessel structure characterized by pericyte detachment (Chabottaux et al., 2009). These observations identify MT4-MMP as a cancer cell-derived MMP with pro-angiogenic and pro-metastatic effect that deserves further in-depth investigations.

## The Protective Effects of MMPs and Related Enzymes

**Figure 2:**
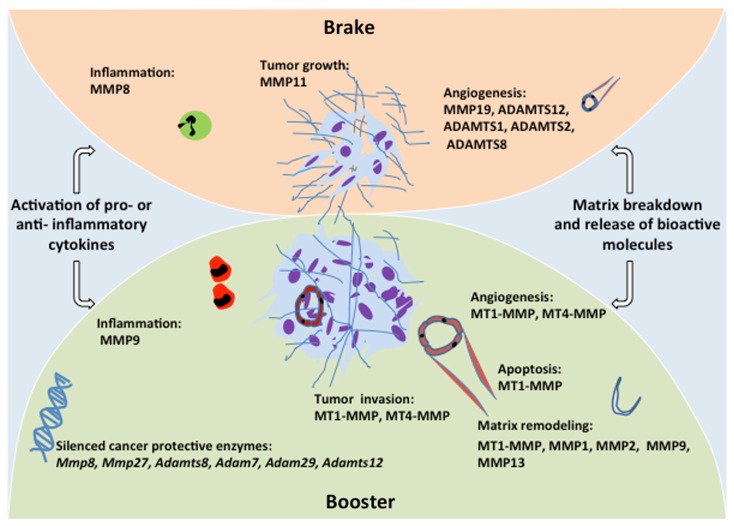
**Schematic representation of the brake and booster functions of metalloproteinases**. Recent advances in genomic and proteomic technologies have increased our knowledge on MMP contributions to different processes associated with tumor development such as tumor growth, angiogenesis, invasion and inflammation. Despite their implication in ECM remodeling and growth factor signaling that favor angiogenesis and boost tumor development, some metalloproteinases exert protective effects that brake the tumor development. Several cancer protective enzymes are silenced through epigenetic and genetic modifications in malignant cancer.

After years of considering MMPs as pro-tumorigenic enzymes, an intriguing observation has prompted re-evaluation of the roles of MMPs in cancer. In fact, MMP8 deficient mice challenged with carcinogens showed a markedly increased susceptibility to tumorigenesis in comparison with corresponding wild-type mice (Balbin et al., 2003). Further histopathological studies demonstrated that sustained inflammation resulting from MMP8-deficiency creates a permissive environment for cancer progression. Importantly, bone marrow transplantation assays in those mutant mice revealed that MMP8-producing neutrophils are sufficient to rescue the anti-tumor protection conferred by this enzyme (Balbin et al., 2003). This study provided the first evidence for a protective role of a MMP family member in tumor progression, which has been further extended to other proteases (Lopez-Otin and Matrisian, 2007) as out-lined below. These findings underline the dual functions of host cells that can either boost the tumor or protect the host toward cancer expansion (Figure 2). In addition, MMP8 downregulation in non-metastatic cells increases their metastatic potential (Montel et al., 2004; Gutierrez-Fernandez et al., 2008), and high MMP8 levels in human carcinomas correlate with lower metastasis incidence and a better prognosis to patients with breast or oral cancer (Decock et al., 2007; Korpi et al., 2008). Such anti-tumor effects or dual functions with protective roles in specific circumstances have been extended to other proteinases including MMP11, MMP12, MMP19, MMP26 (Lopez-Otin and Matrisian, 2007; Lopez-Otin et al., 2009). Furthermore, we reported that ADAMTS-12 exhibits anti-tumorigenic properties by modulating the Ras-dependent ERK pathway (Llamazares et al., 2007). A knock-out mouse strain in which the *Adamts-12* gene is deleted (*Adamts-12^−/−^*) has been established to elucidate the *in vivo* functions of ADAMTS-12 (El Hour et al., 2010). A protective effect of host cell-derived ADAMTS-12 is seen when different *in vivo* models of angiogenesis (malignant keratinocyte transplantation, Matrigel plug, and aortic ring assays) are applied to these knock-out mice. In the absence of ADAMTS-12, both the angiogenic response and tumor invasion into host tissue are increased. This finding is in line with the anti-angiogenic functions reported for other ADAMTS family members such as ADAMTS-1, ADAMTS-2, and ADAMTS-8 (Lee et al., 2006; Rodriguez-Manzaneque et al., 2009; Dubail et al., 2010).

Interestingly, recent large-scale genomic studies have explored the possibility that metalloproteinases could be genetically or epigenetically altered in various human malignant tumors. It appears that human melanomas are frequently associated with mutations in *Mmp8* and *Mmp27* genes leading to loss-of-function and enhanced progression of the cancer (Palavalli et al., 2009). Similarly, somatic mutations are found in *Adamts-15* (Viloria et al., 2009) and *Adamts-18* (Wei et al., 2010) in human colorectal cancer and melanoma samples, respectively. Likewise, *Adam7* and *Adam29* genes are frequently mutated in melanoma (Wei et al., 2010). These findings of tumor-specific mutations, likely to affect tumor cell behavior, implicate these genes as drivers in human cancers and underscore the necessity to revisit the initial concept that alteration of proteinase expression was secondary to transcriptional changes rather than genetic mutations. Beyond somatic mutations, several of the *ADAMTS* genes are epigenetically silenced in various cancers (Moncada-Pazos et al., 2009). The *Adamts-12* promoter is hypermethylated in cancer cell lines and tumor tissues leading to reduced production of ADAMTS-12 (Moncada-Pazos et al., 2009) that exerts anti-tumorigenic effect (Cal et al., 2002). Remarkably, this epigenetic silencing in the tumor cells is associated with a concurrent overexpression of ADAMTS-12 in the stromal compartment (Moncada-Pazos et al., 2009) where it exerts an anti-angiogenic effect (El Hour et al., 2010). These findings suggest that fibroblasts or more likely specific subsets of fibroblasts might react to the presence of tumor cells by overexpressing tumor-inhibiting enzymes. These data provide a strong support for the concept that several proteinases have the ability to apply a brake on cancer cells and protect the host toward cancer progression (Figure 2). Furthermore, they underline the complexity of the tumor-host interface that deserves further in-depth investigation.

## Conclusion and Perspectives

The emerging picture arising from these studies reveals a complex interplay between tumor-derived proteases produced in cancer cells and tumor associated stromal cells, the surrounding cells and the ECM. Tumor cells acquire some of the required properties for growth and invasion by the specific modification of the tumor microenvironment. However, due to the complex nature of these interactions, it is only by altering specific components of this network that it has been possible to identify proteases with pro-tumorigenic or pro-metastatic functions, as well as proteases with tumor-defying properties. The recent identification of recurrently mutated proteases in melanoma and colorectal cancer highlights the growing list of metalloproteinases with protective functions against tumor development. Nevertheless, the mechanisms by which these proteases exert their pro- or anti-tumorigenic properties at the molecular level are largely unknown and represent a challenging issue for the near future. In fact, several MMPs, such as MMP9 or MMP12, might have dual roles either promoting or suppressing tumorigenesis depending on the type of cell in which they are expressed. Given that MMP family members can exert promoting or protective effects and that some individual MMPs can display opposite roles in different cancer types or phases of progression, a required step toward personalized cancer therapy is now the identification of the most appropriate MMP(s) to be targeted in each case. Discerning which MMP(s) to target and when to inhibit are major issues that are facing researchers in the field. In addition, the design of highly selective MMP inhibitors is mandatory to overcome the failure of broad spectrum MMP inhibitors in clinical trials (Table 1). In this context, novel strategies are emerging to generate new specific synthetic inhibitors or neutralizing antibodies (Table 1). Hopefully, the clarification of these questions will finally result in clinical introduction of inhibitors of selected matrix-remodeling enzymes as new components of anticancer therapies.

**Table 1:** **Lessons from the past, present advances, and future challenges for MMP inhibition in cancer**.

Strategies applied	Lessons learnt
**PAST**	
Design of broad spectrum MMP inhibitors (MMPIs) in the decade of 1990’s:	First clinical trials era:
(non-exhaustive list)	No significant evidence of efficacy, and even adverse effects
Zinc-binding MMPIs Mechanism-based MMPIs	Disconnection between promising preclinical studies and clinical trials, most of them being conducted in patients with late stage tumors
Chemically modified tetracycline	Coussens et al. (2002), Overall and Lopez-Otin (2002), Fingleton (2003), Kruger et al. (2010) Synthesized peptides	
Shark cartilage extracts	
Kleifeld et al. (2001), Hu et al. (2007), Devel et al. (2010)	
**PRESENT**	
Novel strategies to generate selective MMPIs:	Era of MMP complexity elucidation:
(non-exhaustive list)	MMPs belong to a protease network (*protease degradome*)
Specific zinc-binding MMPIs	MMPs as cell regulators beyond matrix degradating enzymes
MMPIs without zinc-binding groups	MMPs with intracellular activities
Neutralizing antibodies toward recombinant enzymes Neutralizing antibodies toward catalytic zinc complex	MMPs as builders of the tumor microenvironment in primary and secondary sites (i.e., inflammation, angiogenesis, lymphangiogenesis)
Non-catalytic hemopexin domain (PEX) inhibitors	MMPs with opposite functions depending on cancer type/stage
Humanizing neutralizing monoclonal antibodies raised in	MMPs with tumor suppressive functions
MMP knock-out mice Devel et al. (2006, 2010), Devy et al. (2009), Remacle et al. (2012), Sela-Passwell et al. (2012)	Lopez-Otin et al. (2009), Cauwe and Opdenakker (2010), Fingleton and Lynch (2010), Kruger et al. (2010), Rodriguez et al. (2010), Hua et al. (2011), Schelter et al. (2011a,b), Sounni et al. (2011), Detry et al. (2012)	
**FUTURE**	
Toward new therapeutic approaches:	Challenging issues:
Personalized therapy using selective MMPIs combined with other therapies, including kinase inhibitors	Design of efficient selective inhibitors Design of appropriate clinical trials and endpoints given the fact that MMP inhibitors are expected to be efficient at early stages Lopez-Otin and Hunter (2010)	
	Identification of biomarkers with added values for clinical practice to predict or monitor drug response
	Define which patients will benefit from a specific anti-MMP drug and at which disease stage
	Fingleton (2007, 2008), Hu et al. (2007), Zucker and Cao (2009), Cauwe and Opdenakker (2010), Decock et al. (2011), Hua et al. (2011)

## Conflict of Interest Statement

The authors declare that the research was conducted in the absence of any commercial or financial relationships that could be construed as a potential conflict of interest.
